# Sports Nutrition Ingredients and Governance, Exercise Training, and Sport Technology

**DOI:** 10.1007/s40279-023-01958-2

**Published:** 2023-11-27

**Guest Editor**

Lawrence L. Spriet

Human Health and Nutritional Sciences,

University of Guelph,

Guelph, ON,

Canada

lspriet@uogelph.ca

**Disclosures**: This supplement is supported by the Gatorade Sports Science Institute (GSSI), a division of PepsiCo, Inc.

**Guest Editor Conflicts of Interest**: This supplement was guest edited by Dr. Lawrence L. Spriet, who attended a meeting of the GSSI Expert Panel in October 2022 and received honoraria from GSSI for his participation in the meeting and the writing of the preface for the supplement. Dr. Spriet received no honorarium for guest editing the supplement. Dr. Spriet has no potential conflicts of interest that are directly relevant to the content of this supplement.

